# The effect of titanium dioxide nanoparticles on pulmonary surfactant function and ultrastructure

**DOI:** 10.1186/1465-9921-10-90

**Published:** 2009-09-30

**Authors:** Carsten Schleh, Christian Mühlfeld, Karin Pulskamp, Andreas Schmiedl, Matthias Nassimi, Hans D Lauenstein, Armin Braun, Norbert Krug, Veit J Erpenbeck, Jens M Hohlfeld

**Affiliations:** 1 Fraunhofer Institute of Toxicology and Experimental Medicine, Division of Immunology, Allergology and Airway Research, Nikolai-Fuchs-Str. 1, 30625 Hannover, Germany; 2 Department of Respiratory Medicine, Hannover Medical School, Germany; 3 Institute of Anatomy, Division of Histology, University of Bern, Switzerland; 4 Forschungszentrum Karlsruhe, Institute of Toxicology and Genetics, Department of Molecular and Environmental Toxicology, Germany; 5 Institute of Functional and Applied Anatomy, Hannover Medical School, Germany; 6 Department of Pharmaceutics, Technical University Carolo-Wilhelmina at Braunschweig, Germany

## Abstract

**Background:**

Pulmonary surfactant reduces surface tension and is present at the air-liquid interface in the alveoli where inhaled nanoparticles preferentially deposit. We investigated the effect of titanium dioxide (TiO_2_) nanosized particles (NSP) and microsized particles (MSP) on biophysical surfactant function after direct particle contact and after surface area cycling *in vitro*. In addition, TiO_2 _effects on surfactant ultrastructure were visualized.

**Methods:**

A natural porcine surfactant preparation was incubated with increasing concentrations (50-500 μg/ml) of TiO_2 _NSP or MSP, respectively. Biophysical surfactant function was measured in a pulsating bubble surfactometer before and after surface area cycling. Furthermore, surfactant ultrastructure was evaluated with a transmission electron microscope.

**Results:**

TiO_2 _NSP, but not MSP, induced a surfactant dysfunction. For TiO_2 _NSP, adsorption surface tension (γ_ads_) increased in a dose-dependent manner from 28.2 ± 2.3 mN/m to 33.2 ± 2.3 mN/m (p < 0.01), and surface tension at minimum bubble size (γ_min_) slightly increased from 4.8 ± 0.5 mN/m up to 8.4 ± 1.3 mN/m (p < 0.01) at high TiO_2 _NSP concentrations. Presence of NSP during surface area cycling caused large and significant increases in both γ_ads _(63.6 ± 0.4 mN/m) and γ_min _(21.1 ± 0.4 mN/m). Interestingly, TiO_2 _NSP induced aberrations in the surfactant ultrastructure. Lamellar body like structures were deformed and decreased in size. In addition, unilamellar vesicles were formed. Particle aggregates were found between single lamellae.

**Conclusion:**

TiO_2 _nanosized particles can alter the structure and function of pulmonary surfactant. Particle size and surface area respectively play a critical role for the biophysical surfactant response in the lung.

## Background

High amounts of ambient particulate matter (PM) exist in our atmosphere, and it is known that a high proportion of these particles are nanosized particles (NSP) with a diameter of ≤ 100 nm. NSP can be found in the air as a result of combustion processes such as automobile engines and fires. In addition, the rapidly developing field of nanotechnology is becoming a potential source for human exposure to NSP. Titanium dioxide (TiO_2_) NSP e.g. are widely produced for industrial processes since several years [1].

Importantly, PM exposure is linked with the occurrence of cardio-respiratory disease as well as mortality [2,3]. Epidemiological and experimental data suggest a relationship between PM and e.g. asthma [4], chronic obstructive pulmonary disease [5], and cystic fibrosis [6,7]. Unfortunately, the exact mechanism by which PM induces or aggravates airway disease is still unknown.

Dependent on their size, particles preferentially deposit in different compartments of the lung. Importantly, most of the nanosized particles have a high alveolar deposition rate [8]. In the alveoli, these particles come into contact with the pulmonary surfactant layer that covers the entire alveolar region. Surfactant decreases the surface tension at the air-liquid interface and thereby prevents alveolar collapse. Surface activity is mainly accomplished by surfactant phospholipids and the specific surfactant proteins (SP)-B, and -C. Morphologically, surfactant exists in different subfractions. The surface active fraction consists of lamellar bodies and tubular myelin whereas the less surface active fraction is comprised of unilamellar vesicles. By ultracentrifugation, lamellar bodies and tubular myelin can be pelleted and are thereby called large aggregates (LA). In contrast, unilamellar vesicles remain in the supernatant and are defined as small aggregates (SA). Conversion of LA into SA occurs during respiration [9].

It has been demonstrated that particles of anthropogenic origin are able to directly interact with pulmonary surfactant components [10-13]. Further, it has been shown that nanosized particles can disturb surfactant function [14,15]. However, a systematic comparison of nanosized and microsized particles (MSP) of different composition has not been made. Moreover, it is unclear whether particle-surfactant interactions during dynamic conditions of surface area cycling aggravate the biophysical surfactant dysfunction. Therefore, we investigated the effect of increasing concentrations of TiO_2 _NSP and TiO_2 _MSP, as model particles, on pulmonary surfactant function by means of a pulsating bubble surfactometer both under native conditions and following surface area cycling. For comparison reasons, the effect on surfactant function was investigated for nanosized and microsized polystyrene particles as well as for quartz particles. Furthermore, we studied the effect of nanosized TiO_2_particles on surfactant ultrastructure by transmission electron microscope (TEM). In order to elaborate on the in-vivo relevance, rats were exposed to TiO_2 _NSP versus TiO_2 _MSP, lungs were fixed and lung tissue blocks were prepared for electron microscopy. The ultrastructure and distribution of the different subtypes of intra-alveolar surfactant was observed.

## Methods

### Particles

Nanosized and microsized titanium dioxide particles (Alfa Aesar, Karlsruhe, Germany; product numbers: 44689 & 42681) were used in this study. For comparison, polystyrene particles (Micromod, Rostock-Warnemuende, Germany), Sikron SF 800 quartz particles (Quarzwerke, Frechen, Germany) as well as citrate coated nanosized gold particles (Plano, Wetzlar Germany: product number: EM.GC5) were studied [for details see additional file 1]. Particle stock solutions were prepared in sterilized bidistilled water at a concentration of 25 mg/ml or 50 mg/ml. Particles were sonicated prior to each experiment.

### Acute Effects on Biophysical Surfactant Function

A natural porcine surfactant preparation (Curosurf^®^, Asche Chiesi, Hamburg, Germany) was used as a standard and was adjusted to 1.5 mg/ml phospholipids in Ringer's solution. Particles at increasing concentrations were added (50 μg/ml - 500 μg/ml) and biophysical surfactant function was assessed with a pulsating bubble surfactometer (PBS) (Electronetics, Buffalo, NY, USA) as described below.

### Surface Area Cycling

Surface area cycling is a standardized method to simulate the *in vivo *conversion of surface active surfactant subtypes (lamellar bodies, tubular myelin) to inferior surfactant subtypes (unilamellar vesicles) *in vitro *[16-20]. We measured the biophysical surfactant function following surface area cycling in the presence or absence of particles in order to assess the effect of particles during the conversion process. Curosurf^® ^was adjusted to 1.5 mg/ml phospholipids in ringer solution with or without particles in increasing concentrations (50 μg/ml - 500 μg/ml). Aliquots were placed in 12 × 75 mm capped plastic tubes (Falcon 2058) and rotated end over end for 8 hours at 0.43 Hz and 37°C in the dark. Thereby, surface area changed from 1.1 cm^2 ^to 9 cm^2 ^twice per cycle. After surface area cycling biophysical surfactant function was measured in a pulsating bubble surfactometer as described below.

### Surface Activity Evaluated with the Pulsating Bubble Surfactometer

Surface activity of pulmonary surfactant was measured with a PBS. Forty μl of the surfactant mixture were filled into the sample chamber. The surface tension used for statistical analysis of this study was the value at minimum bubble size (γ_min_) registered after 330 seconds of pulsation at a rate of 20 cycles/min and a temperature of 37°C. In addition, adsorption surface tension (γ_ads_) was evaluated by determining surface tension 10 s after formation of a bubble under static bubble conditions. All data were digitalized and recorded by computer. All assays were performed in duplicate and the mean value was reported. The PBS was calibrated and checked with reference substances for proper operation before starting the measurements on each day.

### Transmission Electron Microscope

Surfactant was fixed in Eppendorf tubes with 1.5% glutaraldehyde and 1.5% paraformaldehyde in 0.15 M Hepes buffer. The samples were stored in the fixative for 1 hour at room temperature and at least 24 hours at 4°C. Afterwards, samples were centrifuged at 10,000 *g *to obtain a surfactant pellet. After several washings in buffer, the samples were subsequently postfixed in osmium tetroxide and half-saturated aqueous uranyl acetate, dehydrated in an ascending acetone series and embedded in Epon at 60°C. The Eppendorf cups were removed and ultrathin 50 nm sections were cut using an ultramicrotome. The sections were analyzed with a Philips CM12 transmission electron microscope (FEI Co. Philips Electron Optics, Zürich, Switzerland).

### Exposure of rats to particles and assessment of surfactant ultrastructure

Female Wistar rats (162 - 200 g) were randomly exposed once for 6 hours to either TiO_2 _NSP (P25; Evonik Degussa, Essen, Germany), TiO_2 _MSP (Bayertitan T, Bayer, Leverkusen, Germany), or clean air, respectively (n = 5 per group). The exposure atmosphere was adjusted to either 10 mg TiO_2 _MSP/m^3 ^or 25 mg TiO_2 _NSP/Na_2_HPO_4_/m^3 ^(60% Na_2_HPO_4; _40% TiO_2 _NSP). Since the TiO_2 _MSP and TiO_2 _NSP/Na_2_HPO_4 _droplets in the atmosphere were approximately of the same size, a similar alveolar deposition rate of 60 μg TiO_2 _particles per animal was accomplished [21]. Rats were sacrificed by pentobarbital overdose at the end of the exposure and the lungs were perfusion-fixated as described before [22]. Surfactant ultrastructure was assessed on ultrathin sections by electron microscopy and surfactant subtype conversion was studied semiquantitatively.

### Statistical Analysis

Values are given as means ± SEM. Statistical analysis was performed using GraphPad Prism^®^, Version 4.03. The one-way ANOVA was used for statistical analysis of the data. A Bonferroni correction was used throughout. P values < 0.05 were considered to be significant.

## Results

### Direct Effect of Particles on Pulmonary Surfactant Function

**Figure 1:**
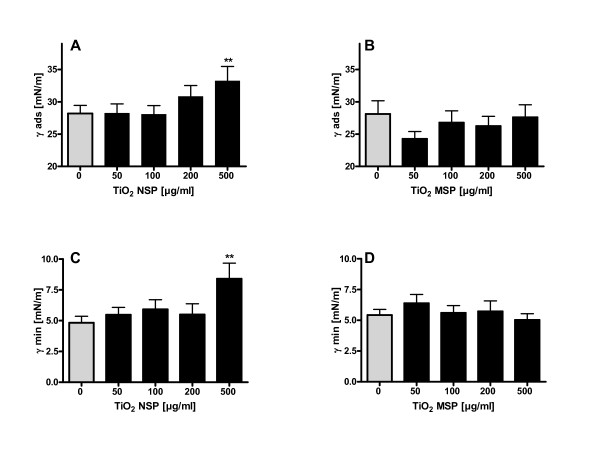
**Surface activity evaluated with the pulsating bubble surfactometer**. A) Adsorption surface tension (γ_ads_) after incubation with TiO_2 _nanosized particles (NSP) at a static bubble condition. B) Influence of TiO_2 _microsized particles (MSP) on γ_ads _C) Influence of TiO_2 _NSP on surface tension at minimal bubble size (γ_min_) during pulsation D) γ_min _after incubation with TiO_2 _MSP. Values are given as means of at least 4 experiments ± SEM. ** indicates p values < 0.01 compared with the control at 0 μg/ml particle concentration.

To assess the direct effect of particles on surfactant function, surface tension was measured after addition of particles. Pure surfactant showed an adsorption surface tension of ~28 mN/m and addition of TiO_2 _NSP in concentrations up to 200 μg/ml did not affect this active surface tension (Figure 1A). However, further increase of the particle dose up to 500 μg/ml led to a significant increase in adsorption surface tension to 33.2 ± 2.3 mN/m. In contrast, surface tension was unaffected by treatment with the same mass concentrations of TiO_2 _MSP (Figure 1B). TiO_2 _NSP slightly increased γ_min _from ≤ 5 mN/m, which denotes active surfactant, up to 8.4 ± 1.3 mN/m at 500 μg/ml (Figure 1C). Again, TiO_2 _MSP showed no effect on surface tension in this concentration range (Figure 1D). However, at very high particle concentrations of TiO_2 _MSP (~10 mg/ml) that deliver a similar surface area compared to TiO_2 _NSP γ_min _increased to 15.9 ± 1.3 mN/m (n = 6, p < 0.01).

As for the TiO_2 _particles similar results were observed for the other particles. Whereas polystyrene NSP significantly increased adsorption surface tension for 500 μg/ml, polystyrene and quartz MSP did not influence the surface tension up to a concentration of 500 μg/ml (Table 1). In addition, nanosized polystyrene particles increased surface tension at minimum bubble size significantly at 500 μg/ml up to 6.8 ± 1.2 mN/m, whereas microsized particles did not influence surfactant function in this concentration range (Table 1). Again, MSP (Quartz) at a very high concentration (~10 mg/ml) that deliver a similar surface area compared to TiO_2 _NSP increased γ_min _to 15.5 ± 1.8 mN/m (n = 5, p < 0.05).

**Table 1:** Surface activity evaluated with the pulsating bubble surfactometer.

	Polystyrene NSP	Polystyrene MSP	Quartz MSP
**Direct effects - γ _ads _[mN/m]**			

**0 μg/ml**	29.2 ± 1.5	28.9 ± 2.7	25.9 ± 1.5
**50 μg/ml**	28.3 ± 1.4	28.3 ± 2.2	25.0 ± 1.6
**100 μg/ml**	30.8 ± 2.2	28.6 ± 2.3	25.0 ± 2.0
**200 μg/ml**	32.4 ± 2.1	30.1 ± 3.0	26.3 ± 2.9
**500 μg/ml**	34.1 ± 2.2**	28.3 ± 2.3	25.2 ± 1.5

**Direct effects - γ _min _[mN/m]**			

**0 μg/ml**	4.0 ± 0.9	2.4 ± 1.2	4.9 ± 0.5
**50 μg/ml**	5.1 ± 0.9	2.2 ± 0.9	4.1 ± 0.9
**100 μg/ml**	4.0 ± 1.0	2.8 ± 1.8	4.6 ± 0.8
**200 μg/ml**	4.7 ± 1.1	3.1 ± 1.3	4.1 ± 0.9
**500 μg/ml**	6.8 ± 1.2**	3.0 ± 1.5	5.0 ± 0.8

**Surface area cycling - γ _ads _[mN/m]**			

**Control**	27.1 ± 1.5	27.4 ± 2.7	28.1 ± 0.8
**0 μg/ml**	45.7 ± 1.0	46.4 ± 1.3	43.7 ± 0.8
**50 μg/ml**	42.4 ± 2.2	43.7 ± 2.2	47.1 ± 0.9
**100 μg/ml**	47.1 ± 1.3	43.1 ± 0.8	45.8 ± 2.8
**200 μg/ml**	44.6 ± 2.1	45.3 ± 2.3	45.4 ± 3.1
**500 μg/ml**	51.4 ± 0.9*	42.7 ± 1.7	48.4 ± 1.5

**Surface area cycling - γ _min _[mN/m]**			

**Control**	1.1 ± 0.2	1.1 ± 0.2	1.2 ± 0.4
**0 μg/ml**	1.2 ± 0.2	1.1 ± 0.2	1.7 ± 0.4
**50 μg/ml**	2.1 ± 0.4	1.1 ± 0.4	0.9 ± 0.3
**100 μg/ml**	1.5 ± 0.5	1.2 ± 0.2	2.1 ± 0.9
**200 μg/ml**	6.3 ± 2.8	1.8 ± 0.5	2.8 ± 0.7
**500 μg/ml**	17.5 ± 1.4***	1.9 ± 0.5	1.0 ± 0.3

Direct effects were measured immediately after addition of particles, effects after surface area cycling were measured following 8 hour rotation at 0.43 Hz with or without particles. Adsorption surface tension (γ_ads_) was obtained from the value of a static bubble. Surface tension at minimal bubble size (γ_min_) was recorded during pulsation. Values are given as means ± SEM of at least 4 experiments. * indicates p values < 0.05; ** indicates p values < 0.01; *** indicates p values < 0.001; all compared with the control without particles (0 μg/ml). In case of surface area cycling, the 0 μg/ml control was rotated for 8 hours at 0.43 Hz. NSP - nanosized particles; MSP - microsized particles.

Furthermore, we tested commercially available gold NSP with citrate coating (5 nm) in single experiments. At 200 μg/ml and 500 μg/ml, gold NSP increased γ_min _to 7.7 ± 2.8 and 13.2 ± 5.3, respectively (n = 4).

### Effects of Particles Following Surface Area Cycling

**Figure 2:**
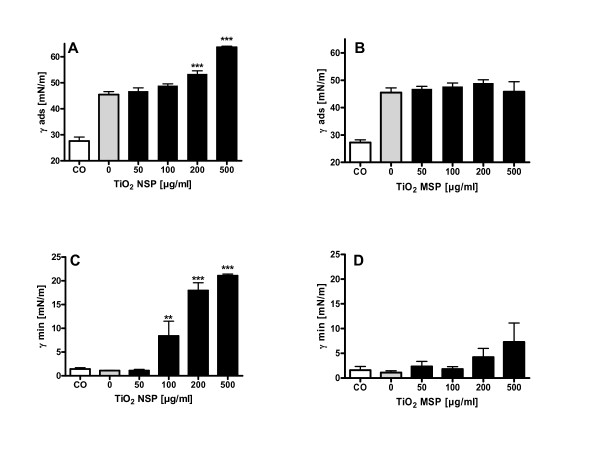
**Surface activity evaluated with the pulsating bubble surfactometer following 8 hour rotation at 0.43 Hz**. A) Influence of TiO_2 _NSP on adsorption surface tension (γ_ads_) at a static bubble condition. B) Influence of TiO_2 _MSP on γ_ads_  C) Influence of TiO_2 _NSP on surface tension at minimal bubble size (γ_min_) during pulsation D) TiO_2 _MSP effect on γ_min_. Values are given as means ± SEM of at least 4 experiments. ** indicates p values < 0.01; *** indicates p values < 0.001; both compared with the rotated 0 μg/ml particle concentration (grey columns). CO/white columns - control surfactant which was placed for 8 hours in an incubator without rotation.

Surface area cycling alone led to an increase in adsorption surface tension from ~28 to ~45 mN/m (Figure 2A and 2B). The presence of TiO_2 _NSP in concentrations of 200 μg/ml and 500 μg/ml during the cycling process led to a further increase of adsorption surface tension to 53.3 ± 1.3 mN/m and 63.6 ± 0.4 mN/m, respectively (Figure 2A). TiO_2 _MSP concentrations up to 500 μg/ml did not affect adsorption surface tension (Figure 2B). The influence of TiO_2 _NSP on surface tension at minimum bubble size was pronounced (Figure 2C). TiO_2 _NSP at 100 μg/ml led to a significant increase in surface tension from 1.1 ± 0.1 mN/m up to 8.4 ± 3.1 mN/m. Further increase of particle dose induced a strong surfactant dysfunction with γ_min _of 18.0 ± 1.6 mN/m and 21.1 ± 0.4 mN/m after incubation with 200 μg/ml and 500 μg/ml TiO_2 _NSP, respectively. TiO_2 _MSP led to a slight but non-significant increase in γ_min _(Figure 2D).

Polystyrene NSP led to a slight increase in adsorption surface tension from 45.7 ± 1.0 mN/ml up to 51.4 ± 0.9 mN/ml which was only significant at a concentration of 500 μg/ml polystyrene NSP (Table 1). All other MSP did not influence adsorption surface tension (Table 1). Surface tension at minimum bubble size was also unaffected by polystyrene MSP and quartz MSP (Table 1), while polystyrene NSP induced a strong surfactant dysfunction at minimum bubble size. Incubation with 500 μg/ml polystyrene NSP during the cycling process led to a surface tension of 17.5 ± 1.4 mN/m (Table 1).

### Influence of Nanosized TiO_2 _Particles on Surfactant Ultrastructure

**Figure 3:**
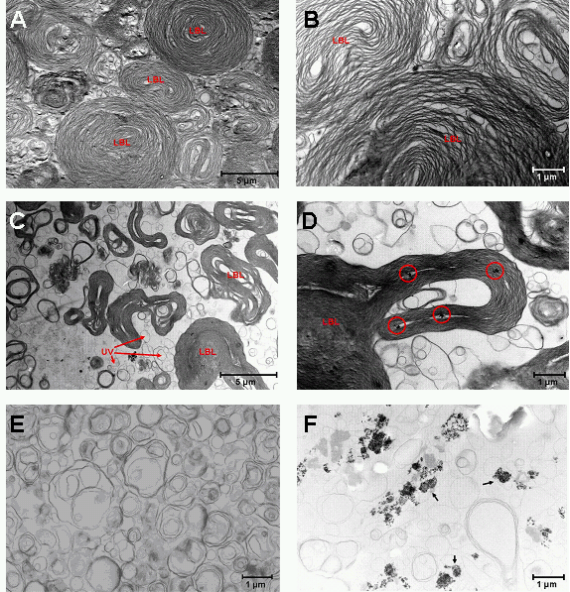
**Representative transmission electron microscope pictures of the surfactant ultrastructure**. A) and B) untreated control surfactant. C) and D) porcine surfactant after addition of 100 μg/ml TiO_2 _nanosized particles. Red circles show small particle aggregates. E) Control surfactant after 8 hours rotation at 0.43 Hz and 37°C. F) Surfactant after 8 hours rotation at 0.43 Hz and 37°C in the presence of 100 μg/ml TiO_2 _nanosized particles. Black arrows show large particle aggregates; lbl - lamellar body like forms; ulv - unilamellar vesicles.

Natural porcine surfactant used in this study consisted mostly of lamellar body-like forms. Unilamellar vesicles were hardly present (Figure 3A and 3B). After addition of 100 μg/ml TiO_2 _NSP, lamellar body-like forms were decreased in size and deformed (Figure 3C). In addition, an increase in the amount of unilamellar vesicles appeared (Figure 3C). Interestingly, small TiO_2 _NSP aggregates accumulated between lamellae of the lamellar body-like forms (Figure 3D). Rotation of the pure surfactant in the absence of particles readily led to a conversion of lamellar body-like forms to unilamellar vesicles (Figure 3E). Rotation in the presence of TiO_2 _NSP did not further change subtype conversion (Figure 3F). However, large TiO_2 _aggregates were found after rotation (Figure 3F). These aggregates were larger in size than the TiO_2 _aggregates in the non-rotated sample (Figure 3D).

### Effect of inhaled particles on surfactant ultrastructure in rats

Semiquantitative analysis of intra-alveolar active (tubular myelin and lamellar bodies) and inactive surfactant subtypes (unilamellar vesicles) did not differ between both groups. There were no signs of alveolar oedema or inflammation.

## Discussion

The present data show that nanosized particles, but not microsized particles, induce a dysfunction of pulmonary surfactant. Nanosized titanium dioxide as well as nanosized polystyrene particles at high concentrations can induce a slight pulmonary surfactant dysfunction *in vitro*. Interestingly, surface area cycling *in vitro *aggravated the surfactant dysfunction induced by nanoparticles, both by TiO_2 _NSP and by polystyrene NSP. In addition, biophysical alterations of pulmonary surfactant by TiO_2 _NSP were accompanied by changes of the surfactant ultrastructure indicating increased surfactant subtype conversion.

A direct interaction between particles and the surfactant constituents is the most likely explanation for the observed surfactant dysfunction. It is well known that phospholipids bind to particles [10,14,23] and to TiO_2 _structures [24,25]. In this respect, surface area seems to be the major determinant of the observed biophysical and ultrastructural changes. Accordingly, particles with the highest surface area - TiO_2 _NSP and also reference polystyrene NSP - induced the most prominent alterations. Microsized particles with a relatively low surface area did not induce a surfactant dysfunction in our study.

In separate experiments, we compared equal surface areas by testing very high microparticle mass concentrations. With concentrations of ~10 mg/ml TiO_2 _MSP and quartz MSP, we observed a strong surfactant dysfunction. However, the experimental conditions were limited because microsized particles at this very high concentration aggregated and rapidly sedimented to the bottom of the test capillary. By this segregation, the phospholipid concentration was not stable which limits the comparison of NSP and MSP at similar surface areas.

Bakshi and coworkers demonstrated a potent pulmonary surfactant dysfunction at low concentrations of ~2 μg/ml gold nanoparticles [14]. In contrast, much higher concentrations of TiO_2 _NSP were required to induce an increase of surface tension in our experiments. In addition, the degree of surfactant dysfunction was less with TiO_2 _NSP in our study compared to the gold nanoparticles used by Bakshi et al. Differences in 1) the measuring system, 2) the surfactant preparation and concentration, or 3) the nanoparticles themselves might account for the discrepancy. Both, the pulsating bubble surfactometer (PBS) and the captive bubble surfactometer (CPS) are able to evaluate low surface tensions [26] while the CPS is regarded to yield even lower surface tensions [27] which makes differences in the device an unlikely explanation. Regarding surfactant preparation and concentration, we used Curosurf^®^, a natural surfactant derived from minced porcine lungs [28] while a semisynthetic surfactant composed of two phospholipids plus SP-B was used by Bakshi. It is unlikely that differences in the surfactants are solely responsible for the different effects seen with gold nanoparticles and TiO_2 _NSP. Both surfactants have been demonstrated to have excellent surface activity and to achieve very low surface tensions under compression at the concentrations used. The most likely explanation for the potent dysfunction in the study by Bakshi seems related to the material properties (size/surface) of the gold nanoparticles. Since the gold NSP had citrate groups on their surface, aggregation is mostly avoided [29]. In contrast, pure TiO_2 _nanoparticles highly aggregate. Although the surface area is not known for the gold NSP from Bakshis study, it is likely that the surface area per mass unit is higher for the citrate coated gold NSP than for the TiO_2 _NSP. This could explain the more potent induction of surfactant dysfunction by gold NSP compared to TiO_2 _NSP because surfactant components could be bound to the large gold nanoparticle surface area making them unavailable for lowering surface tension at the air-liquid interface.

In an attempt of direct comparison between TiO_2 _NSP and the gold nanoparticles by Bakshi (~15 nm), we tested commercially available gold NSP with citrate coating (5 nm) in single experiments. Interestingly, at equal mass the surfactant dysfunction by gold NSP was stronger compared with TiO_2 _NSP. However, the dysfunction was less compared with data from Bakshi et al., but this discrepancy can be accounted to differences in surface area of the gold NSP or the surfactant preparations used in both studies.

The *in vivo *conversion of surface active LA to inferior SA can be simulated *in vitro *by surface area cycling [16]. By this technique, the impact of meconium, serum proteins, or surfactant proteins during the surfactant conversion process have been studied [19,30-32]. We assessed the effect of TiO_2 _NSP on the conversion process. Importantly, a dose-dependent increase in surface tension was obtained. Remarkably, this effect was much stronger than the direct biophysical effect of TiO_2 _NSP without cycling. TEM pictures demonstrated that the occurrence of unilamellar vesicles was independent from NSP presence. Possibly, binding of NSP to SP-B and subsequent loss of SP-B from the air-liquid-interface could explain the loss of surface activity following surface area cycling in the presence of NSP. *In vivo*, SP-B becomes cleaved by a serine active carboxylesterase called convertase [33-35]. However, Curosurf^® ^is prepared by chlorofom extraction and hence does not contain convertase [20]. Therefore, intact SP-B should be present in Curosurf^® ^following surface area cycling. We speculate that free SP-B could interact with TiO_2 _NSP which in turn becomes depleted leading to disturbed surfactant function. High ability of SP-B to bind to surfaces during surface area cycling was shown before when binding of SP-B to tube walls was investigated during surface area cycling [18]. Unfortunately, we were not able to provide direct evidence of binding of SP-B to TiO_2 _NSP by TEM due to methodological limitations.

Admittedly, inhaled particles act directly on the surfactant layer at the air-liquid interface and not primarily through the subphase as in our *in vitro *experiments. However, after deposition at the air-liquid interface the particles subsequently become dissolved in the epithelial lining layer and interfere with the dynamic process of phospholipid arrangement at the interface. Therefore, the assay system with the pulsating bubble surfactometer is at least capable to demonstrate differential effects of nanoparticles versus microparticles in phospholipid suspensions when particles interfere with the formation of the surfactant layer from the hypophase. It is very well conceivable that the initial effect of particles might even be greater when they are reaching the interface directly.

Although we have demonstrated that TiO_2 _NSP elicited biophysical and structural changes of surfactant *in vitro*, the *in vivo *relevance has to be scrutinized because the particle concentrations that we found effective *in vitro *can hardly occur *in vivo*. With the human alveolar surface area of ~100 m^2 ^and assuming an average thickness of the alveolar lining fluid of approximately 200 nm [36], the amount of alveolar lining fluid can be assessed as ~20 ml. In accordance, the epithelial lining fluid has been suggested to be 6 ml/L total lung capacity, resulting in 40 ml in man [37]. With the assumption of a particle concentration of 100 μg/m^3^, which can occur in polluted inner cities, and an alveolar deposition rate as high as 50%, the amount of particles deposited per day would be ~360 μg. At steady state, this would result in a concentration of ~10 μg nanoparticles per ml alveolar lining fluid. This particle concentration is far below what has been demonstrated to cause a surfactant dysfunction in our study. In addition, clearance of particles and secretion of newly synthesized surfactant would further improve this particle/surfactant ratio and consequently question whether nanoparticles can cause a surfactant alteration under these conditions *in vivo*. This view is supported by our experimental evidence in rats. Following inhalation of TiO_2 _particles that were aerosolized and adjusted to result in the highest technically possible alveolar deposition of 60 μg particles per animal, surfactant ultrastructure was found unaffected *in vivo*. Assuming an alveolar lining fluid in rats of approximately 70 μL [36], the *in vivo *particle concentration in the epithelial lining fluid would have been approximately 53.5 μg/mL (normalized to 1.5 mg/ml phospholipids and assuming static conditions). Noteworthy, the local concentration at the air-liquid interface was probably much higher suggesting that no changes of surfactant ultrastructure occur *in vivo *under acute maximal TiO_2 _particle exposure.

Although these considerations suggest that the impact of TiO_2 _NSP on surfactant function in the human lung is highly unlikely to cause adverse effects in healthy individuals, in diseased subjects, however, additive effects of NSP on pulmonary surfactant function and ultrastructure have to be taken into account. For example, it has been demonstrated that a pulmonary surfactant dysfunction can be found in various airway diseases like asthma [38], cystic fibrosis [39], or after lung transplantation [40]. In particular, leakage of plasma proteins into the airway lumen is known to induce a surfactant dysfunction [41,42]. Importantly, NSP can induce [43-45] or enhance [46,47] pulmonary inflammation which is accompanied by protein leakage. This in turn could lead to a surfactant dysfunction *in vivo*. Moreover, NSP are able to induce oxidative stress and lipid peroxidation [48-50]. Oxidative stress with lipid peroxidation can induce an increase in surface tension [51,52]. In addition, NSPs emitted by engines are contaminated with alkanes and sulfates [53] and it is known, that eicosane, a specific n-alkane constituent of diesel exhaust NSPs, can affect the biophysical surfactant function [54]. Therefore, nanoparticles might amplify alterations of the pulmonary surfactant system, particularly under predisposed conditions of airway inflammation.

## Conclusion

Taken together, TiO_2 _NSP induce biophysical and structural alterations of pulmonary surfactant *in vitro*. Under dynamic conditions of surface area cycling, this interfering impact is aggravated. Although our data do not suggest that inhalation of nanoparticles cause a significant disturbance of the pulmonary surfactant system *in vivo*, nanoparticles might be detrimental in patients with preexisting airway disease.

## Abbreviations

CPS: Captive bubble surfactometer; CO: Control; DPPC: Dipalmitoylphosphatidylcholine; LA: Large aggregates; lbl: Lamellar body-like forms; MSP: Microsized particles; NSP: Nanosized particles; PBS: Pulsating bubble surfactometer; PM: Particulate matter; SA: Small aggregates; SEM: Standard error of the mean; SP: Surfactant protein; TEM: Transmission electron microscope; TiO_2 _: Titanium dioxide; ulv: Unilamellar vesicles; γ_min_: surface tension at minimum bubble size; γ_ads_: adsorption surface tension

## Competing interests

The authors declare that they have no competing interests.

## Authors' contributions

CS planned the concept and study design, performed the surface area cycling as well as pulsating bubble surfactometer experiments, interpreted the results and wrote major parts of the manuscript. CM performed the electron microscopic analyses of the surfactant ultrastructure and interpreted the results. KP performed the electron microscopic analyses of the particles and interpreted the results. AS performed the electron microscopic analyses of the surfactant ultrastructure and interpreted the results. MN participated in characterization of the particles and interpreted the results. HDL made substantial contributions to the analysis and interpretation of the data. AB made substantial contributions to the analysis and interpretation of the data. NK made substantial contributions to the analysis and interpretation of the data. VJE participated in planning the study design and made substantial contributions to the analysis and interpretation of the data. JMH planned the concept and study design, made substantial contributions to the analysis and interpretation of the data and wrote major parts of the manuscript. All of the authors have critically read the manuscript and approved its submission.

## Supplementary Material

Additional file 1 **Characterization of the particles**. Representative transmission electron microscopic pictures of the particles [see figure S1 in additional file 1] as well as a table with the characteristics of the particles [see table S1 in additional file 1] are shown. Click here for file

## References

[B1] Ellsworth DK Verhulst D Spitler TM Sabacky BJ Titanium nanoparticles move to the marketplace Chemical Innovation 2000 30 30 35

[B2] Dockery DW Pope CA III Xu X Spengler JD Ware JH Fay ME An association between air pollution and mortality in six U.S. cities N Engl J Med 1993 329 1753 1759 10.1056/NEJM199312093292401 8179653

[B3] Wichmann HE Spix C Tuch T Wolke G Peters A Heinrich J Daily mortality and fine and ultrafine particles in Erfurt, Germany part I: role of particle number and particle mass Res Rep Health Eff Inst 2000 5 86 11918089

[B4] Li N Hao M Phalen RF Hinds WC Nel AE Particulate air pollutants and asthma. A paradigm for the role of oxidative stress in PM-induced adverse health effects Clin Immunol 2003 109 250 265 10.1016/j.clim.2003.08.006 14697739

[B5] Kirkham P Rahman I Oxidative stress in asthma and COPD: antioxidants as a therapeutic strategy Pharmacol Ther 2006 111 476 494 10.1016/j.pharmthera.2005.10.015 16458359

[B6] Collaco JM Vanscoy L Bremer L McDougal K Blackman SM Bowers A Interactions between secondhand smoke and genes that affect cystic fibrosis lung disease JAMA 2008 299 417 424 10.1001/jama.299.4.417 18230779 PMC3139475

[B7] Goss CH Newsom SA Schildcrout JS Sheppard L Kaufman JD Effect of ambient air pollution on pulmonary exacerbations and lung function in cystic fibrosis Am J Respir Crit Care Med 2004 169 816 821 10.1164/rccm.200306-779OC 14718248

[B8] Oberdorster G Oberdorster E Oberdorster J Nanotoxicology: an emerging discipline evolving from studies of ultrafine particles Environ Health Perspect 2005 113 823 839 16002369 10.1289/ehp.7339 PMC1257642

[B9] Ikegami M Surfactant catabolism Respirology 2006 11 Suppl S24 S27 10.1111/j.1440-1843.2006.00803.x 16423266

[B10] Mornet S Lambert O Duguet E Brisson A The formation of supported lipid bilayers on silica nanoparticles revealed by cryoelectron microscopy Nano Lett 2005 5 281 285 10.1021/nl048153y 15794611

[B11] Kendall M Fine airborne urban particles (PM2.5) sequester lung surfactant and amino acids from human lung lavage Am J Physiol Lung Cell Mol Physiol 2007 293 L1053 L1058 10.1152/ajplung.00131.2007 17616648

[B12] Hill CA Wallace WE Keane MJ Mike PS The enzymatic removal of a surfactant coating from quartz and kaolin by P388D1 cells Cell Biol Toxicol 1995 11 119 128 10.1007/BF00767497 7583872

[B13] Schleh C Hohlfeld JM Interaction of nanoparticles with the pulmonary surfactant system Inhal Toxicol 2009 21 97 103 10.1080/08958370903005744 19558240

[B14] Bakshi MS Zhao L Smith R Possmayer F Petersen NO Metal nanoparticle pollutants interfere with pulmonary surfactant function in vitro Biophys J 2008 94 855 868 10.1529/biophysj.107.106971 17890383 PMC2186259

[B15] Ku T Gill S Lobenberg R Azarmi S Roa W Prenner EJ Size dependent interactions of nanoparticles with lung surfactant model systems and the significant impact on surface potential J Nanosci Nanotechnol 2008 8 2971 2978 10.1166/jnn.2008.171 18681033

[B16] Gross NJ Narine KR Surfactant subtypes of mice: metabolic relationships and conversion in vitro J Appl Physiol 1989 67 414 421 2759970 10.1152/jappl.1989.67.1.414

[B17] Beatty AL Malloy JL Wright JR Pseudomonas aeruginosa degrades pulmonary surfactant and increases conversion in vitro Am J Respir Cell Mol Biol 2005 32 128 134 10.1165/rcmb.2004-0276OC 15528490

[B18] Inchley K Cockshutt A Veldhuizen R Possmayer F Dissociation of surfactant protein B from canine surfactant large aggregates during formation of small surfactant aggregates by in vitro surface area cycling Biochim Biophys Acta 1999 1440 49 58 10477824 10.1016/s1388-1981(99)00112-2

[B19] Kakinuma R Shimizu H Ogawa Y Effect of meconium on the rate of in vitro subtype conversion of swine pulmonary surfactant Eur J Pediatr 2002 161 31 36 11808878 10.1007/s00431-001-0858-8

[B20] Veldhuizen RA Yao LJ Lewis JF An examination of the different variables affecting surfactant aggregate conversion in vitro Exp Lung Res 1999 25 127 141 10.1080/019021499270349 10188107

[B21] Price OT Asgharian B Miller FJ Cassee FR de Winter-Sorkina R Multiple Path Particle Dosimetry model (MPPD v1.0): A model for human and rat airway particle dosimetry RIVM rapport 650010030 CD-rom publication 2002

[B22] Schmiedl A Hoymann HG Ochs M Menke A Fehrenbach A Krug N Increase of inactive intra-alveolar surfactant subtypes in lungs of asthmatic Brown Norway rats Virchows Arch 2003 442 56 65 12536315 10.1007/s00428-002-0720-z

[B23] Blank F Rothen-Rutishauser BM Schurch S Gehr P An optimized in vitro model of the respiratory tract wall to study particle cell interactions J Aerosol Med 2006 19 392 405 10.1089/jam.2006.19.392 17034314

[B24] Fortunelli A Monti S Simulations of lipid adsorption on TiO2 surfaces in solution Langmuir 2008 24 10145 10154 10.1021/la801787s 18712891

[B25] Jiang C Gamarnik A Tripp CP Identification of lipid aggregate structures on TiO2 surface using headgroup IR bands J Phys Chem B 2005 109 4539 4544 10.1021/jp046042h 16851530

[B26] Putz G Goerke J Taeusch HW Clements JA Comparison of captive and pulsating bubble surfactometers with use of lung surfactants J Appl Physiol 1994 76 1425 1431 8045815 10.1152/jappl.1994.76.4.1425

[B27] Rudiger M Kolleck I Putz G Wauer RR Stevens P Rustow B Plasmalogens effectively reduce the surface tension of surfactant-like phospholipid mixtures Am J Physiol 1998 274 L143 L148 9458812 10.1152/ajplung.1998.274.1.L143

[B28] Bernhard W Mottaghian J Gebert A Rau GA Hardt Poets CF Commercial versus native surfactants. Surface activity, molecular components, and the effect of calcium Am J Respir Crit Care Med 2000 162 1524 1533 11029372 10.1164/ajrccm.162.4.9908104

[B29] Kim T Lee CH Joo SW Lee K Kinetics of gold nanoparticle aggregation: experiments and modeling J Colloid Interface Sci 2008 318 238 243 10.1016/j.jcis.2007.10.029 18022182

[B30] Veldhuizen RA Inchley K Hearn SA Lewis JF Possmayer F Degradation of surfactant-associated protein B (SP-B) during in vitro conversion of large to small surfactant aggregates Biochem J 1993 295 Pt 1 141 147 8216208 10.1042/bj2950141 PMC1134830

[B31] Ueda T Ikegami M Jobe A Surfactant subtypes. In vitro conversion, in vivo function, and effects of serum proteins Am J Respir Crit Care Med 1994 149 1254 1259 8173767 10.1164/ajrccm.149.5.8173767

[B32] Veldhuizen RA Yao LJ Hearn SA Possmayer F Lewis JF Surfactant-associated protein A is important for maintaining surfactant large-aggregate forms during surface-area cycling Biochem J 1996 313 Pt 3 835 840 8611163 10.1042/bj3130835 PMC1216986

[B33] Gross NJ Schultz RM Requirements for extracellular metabolism of pulmonary surfactant: tentative identification of serine protease Am J Physiol 1992 262 L446 L453 1566860 10.1152/ajplung.1992.262.4.L446

[B34] Gross NJ Bublys V D'Anza J Brown CL The role of alpha 1-antitrypsin in the control of extracellular surfactant metabolism Am J Physiol 1995 268 L438 L445 7900825 10.1152/ajplung.1995.268.3.L438

[B35] Ruppert C Pucker C Markart P Schmidt R Grimminger F Seeger W Selective inhibition of large-to-small surfactant aggregate conversion by serine protease inhibitors of the bis-benzamidine type Am J Respir Cell Mol Biol 2003 28 95 102 10.1165/rcmb.4591 12495937

[B36] Bastacky J Lee CY Goerke J Koushafar H Yager D Kenaga L Alveolar lining layer is thin and continuous: low-temperature scanning electron microscopy of rat lung J Appl Physiol 1995 79 1615 1628 8594022 10.1152/jappl.1995.79.5.1615

[B37] Effros RM Feng NH Mason G Sietsema K Silverman P Hukkanen J Solute concentrations of the pulmonary epithelial lining fluid of anesthetized rats J Appl Physiol 1990 68 275 281 10.1063/1.347197 2312470

[B38] Hohlfeld JM Ahlf K Enhorning G Balke K Erpenbeck VJ Petschallies J Dysfunction of pulmonary surfactant in asthmatics after segmental allergen challenge Am J Respir Crit Care Med 1999 159 1803 1809 10351922 10.1164/ajrccm.159.6.9806145

[B39] Griese M Birrer P Demirsoy A Pulmonary surfactant in cystic fibrosis Eur Respir J 1997 10 1983 1988 10.1183/09031936.97.10091983 9311489

[B40] Hohlfeld JM Tiryaki E Hamm H Hoymann HG Krug N Haverich A Pulmonary surfactant activity is impaired in lung transplant recipients Am J Respir Crit Care Med 1998 158 706 712 9730994 10.1164/ajrccm.158.3.9708063

[B41] Ikegami M Jobe A Jacobs H Lam R A protein from airways of premature lambs that inhibits surfactant function J Appl Physiol 1984 57 1134 1142 6209255 10.1152/jappl.1984.57.4.1134

[B42] Holm BA Notter RH Finkelstein JN Surface property changes from interactions of albumin with natural lung surfactant and extracted lung lipids Chem Phys Lipids 1985 38 287 298 10.1016/0009-3084(85)90022-2 3841303

[B43] Sung JH Ji JH Yoon JU Kim DS Song MY Jeong J Lung function changes in Sprague-Dawley rats after prolonged inhalation exposure to silver nanoparticles Inhal Toxicol 2008 20 567 574 10.1080/08958370701874671 18444009

[B44] Niwa Y Hiura Y Sawamura H Iwai N Inhalation exposure to carbon black induces inflammatory response in rats Circ J 2008 72 144 149 10.1253/circj.72.144 18159116

[B45] Ferin J Oberdorster G Penney DP Pulmonary retention of ultrafine and fine particles in rats Am J Respir Cell Mol Biol 1992 6 535 542 1581076 10.1165/ajrcmb/6.5.535

[B46] Inoue K Takano H Yanagisawa R Hirano S Sakurai M Shimada A Effects of airway exposure to nanoparticles on lung inflammation induced by bacterial endotoxin in mice Environ Health Perspect 2006 114 1325 1330 16966083 10.1289/ehp.8903 PMC1570092

[B47] Inoue K Takano H Yanagisawa R Ichinose T Sakurai M Yoshikawa T Effects of nano particles on cytokine expression in murine lung in the absence or presence of allergen Arch Toxicol 2006 80 614 619 10.1007/s00204-006-0075-3 16482471

[B48] Worle-Knirsch JM Kern K Schleh C Adelhelm C Feldmann C Krug HF Nanoparticulate vanadium oxide potentiated vanadium toxicity in human lung cells Environ Sci Technol 2007 41 331 336 10.1021/es061140x 17265967

[B49] Arora S Jain J Rajwade JM Paknikar KM Cellular responses induced by silver nanoparticles: In vitro studies Toxicol Lett 2008 179 93 100 10.1016/j.toxlet.2008.04.009 18508209

[B50] Sayes CM Marchione AA Reed KL Warheit DB Comparative pulmonary toxicity assessments of C60 water suspensions in rats: few differences in fullerene toxicity in vivo in contrast to in vitro profiles Nano Lett 2007 7 2399 2406 10.1021/nl0710710 17630811

[B51] Seeger W Lepper H Wolf HR Neuhof H Alteration of alveolar surfactant function after exposure to oxidative stress and to oxygenated and native arachidonic acid in vitro Biochim Biophys Acta 1985 835 58 67 3924109 10.1016/0005-2760(85)90030-x

[B52] Shelley SA Oxidant-induced alterations of lung surfactant system J Fla Med Assoc 1994 81 49 51 8133236

[B53] Kittelson DB Engines and nanoparticles: A review Journal of Aerosol Science 1998 29 575 588 10.1016/S0021-8502(97)10037-4

[B54] Kanno S Furuyama A Hirano S Effects of eicosane, a component of nanoparticles in diesel exhaust, on surface activity of pulmonary surfactant monolayers Arch Toxicol 2008 82 841 850 10.1007/s00204-008-0306-x 18488198

