# Supplementary material for: The effect of titanium dioxide nanoparticles on pulmonary surfactant function and ultrastructure
Source: Respir Res. 2009 Sep 30;10(1):90. doi: 10.1186/1465-9921-10-90 (PMC2765946; doi:10.1186/1465-9921-10-90)
Supplement: Additional file 1 — Characterization of the particles. Representative transmission electron microscopic pictures of the particles [see figure S1 in additional file 1] as well as a table with the characteristics of the particles [see table S1 in additional file 1] are shown. [file 1465-9921-10-90-S1.DOC]

**Supplemental Information**

**Material & Methods**

**Particle Characterization**

Primary particle size as well as purity specifications were provided by the suppliers. Primary particle sizes were confirmed by transmission electron microscope (TEM) images. The total surface areas of the polystyrene particles was calculated as the product of the particle number and individual surface area. BET (Brunnauer, Emmett, Teller) surface areas of titanium dioxide particles as well as quartz particles were measured by the suppliers.

**Representative Pictures of Particles**

Particles were prepared in ringer solution as for use in the experiments and dropped on 75-mesh Formvar coated copper grids, dried, and analyzed with a Zeiss 109T transmission electron microscope (Oberkochen, Germany).

**Results**

**Particle Characterization**

The primary particle sizes were specified by the suppliers (Table S1) and were confirmed by TEM pictures (Figure S1). In some cases we found individual particles that were smaller than the denoted size range. However, these particles were in the minority. Two different nanosized particles (NSP) were used in this study: TiO_2_ (5 nm) and polystyrene (50 nm). In addition, three different microsized particles (MSP) were used: TiO_2_ (900-1600 nm), polystyrene (1000 nm), as well as quartz (100-16000 nm). As expected, the particles tend to aggregate. However, single particles could be found, too. The highest surface area was found for TiO_2_ NSP (210 m^2^/g) , followed by polystyrene NSP with 114 m^2^/g. The surface areas of the MSP were much lower. Values of 6.0 m^2^/g (quartz), 5.7 m^2^/g (polystyrene), and 4.2 m^2^/g (TiO_2_) were measured (Table S1).

Figure Legends

Figure S1

Representative transmission electron micrograph pictures of TiO_2_ nanosized particles (NSP) (A), TiO_2_ microsized particles (MSP) (B), polystyrene NSP (C), polystyrene MSP (D), as well as quartz MSP (E).

Table S1

Characterization of the particles used in this study. The primary particles sizes are manufacturer's data and were confirmed by transmission electron microscopy. The BET (Brunnauer, Emmett, Teller) surface area from quartz as well as TiO_2_ particles were measured by the manufacturer. The surface areas from the polystyrene particles were calculated as the product of the particle number and individual surface area. d_50%_ median diameter; NSP nanosized particles; MSP microsized particles

Figure S1: Representative TEM Images

A) TiO_2_ NSP B) TiO_2_ MSP


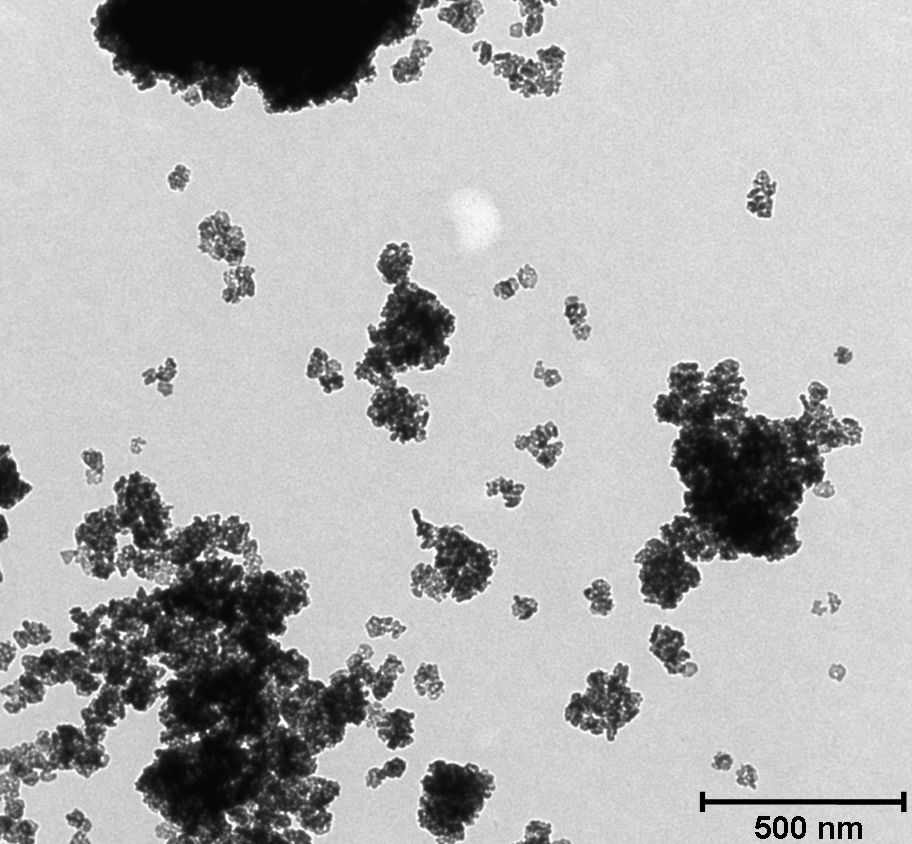

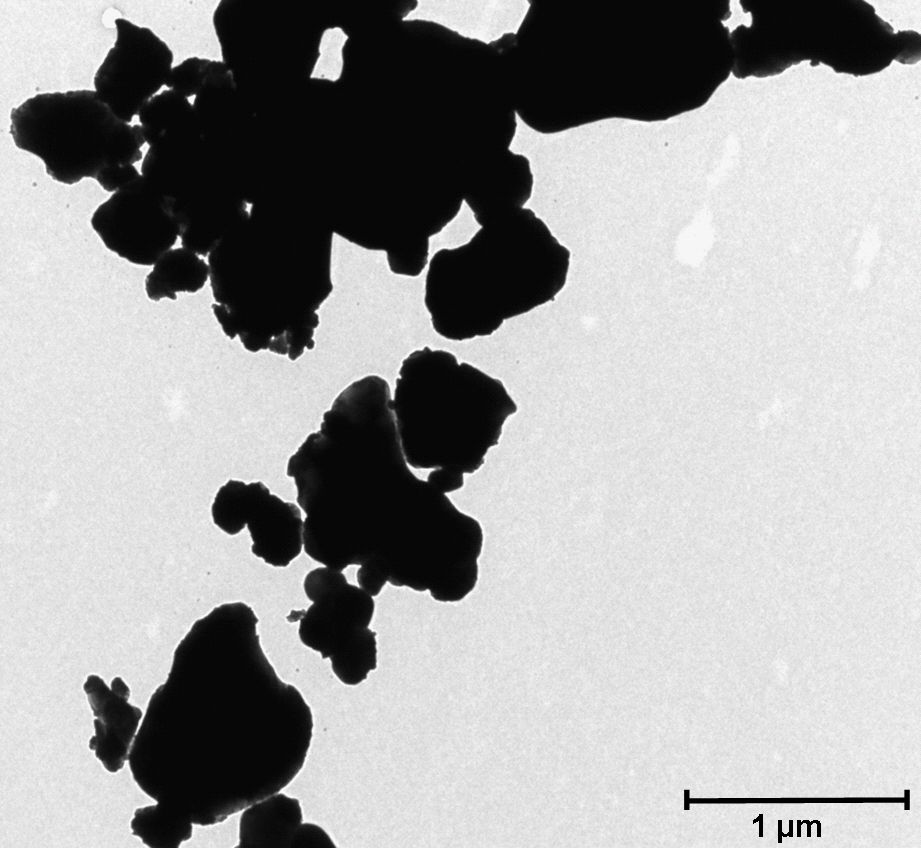


C) Polystyrene NSP D) Polystyrene MSP


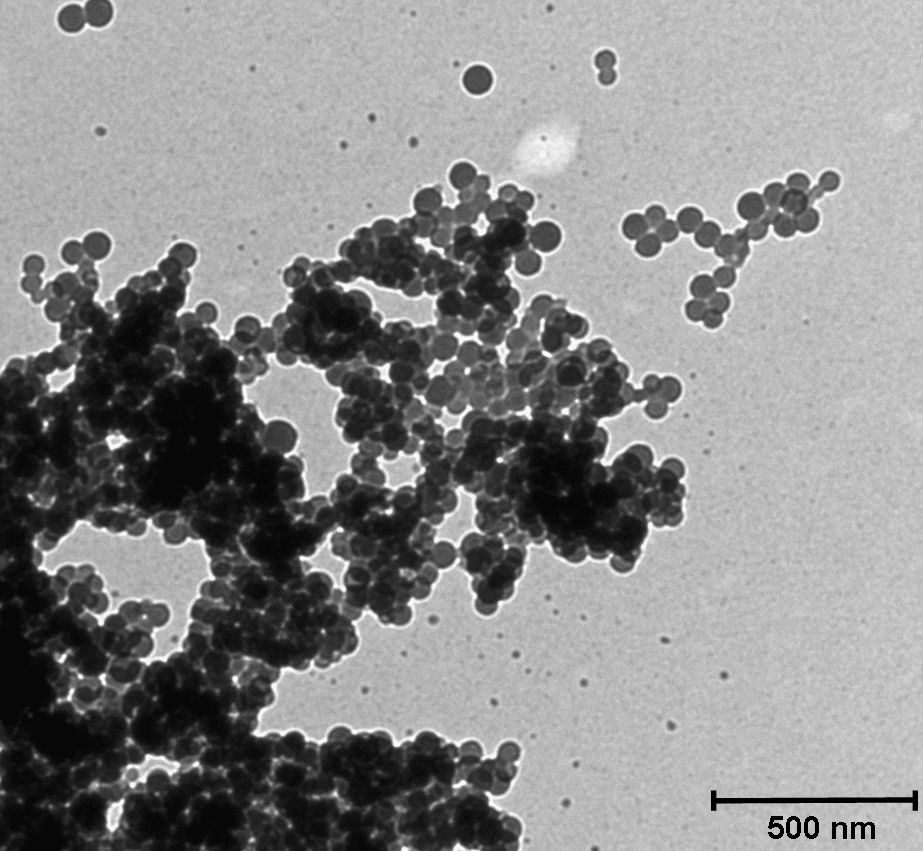

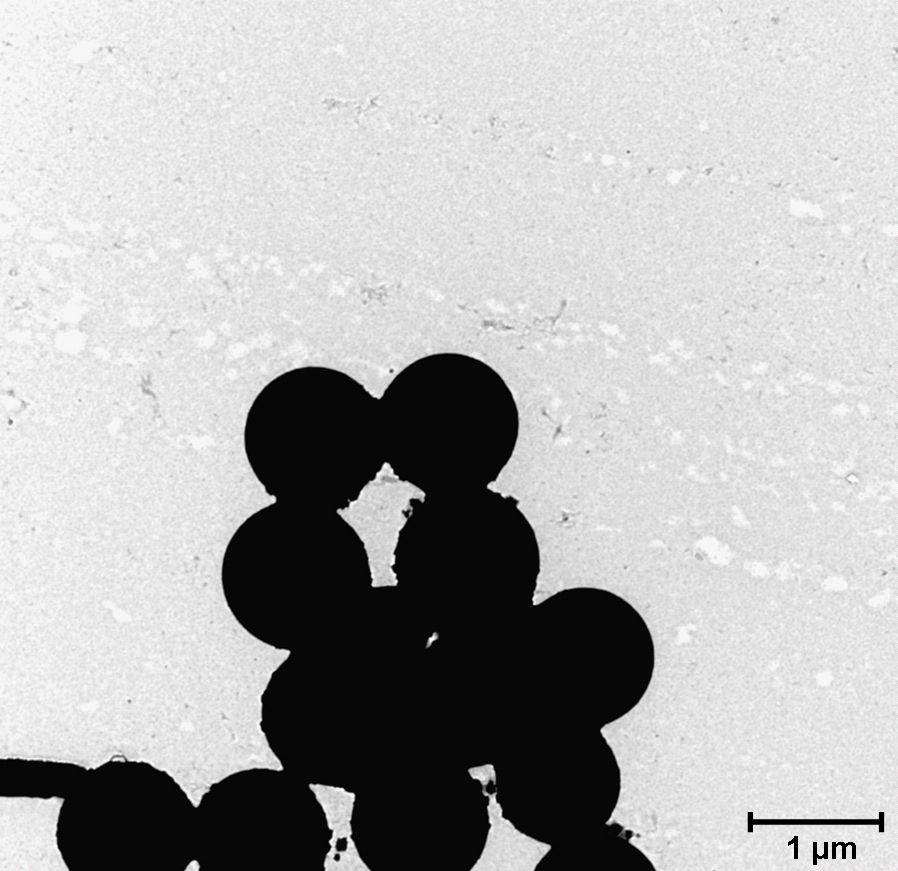


E) Quartz MSP


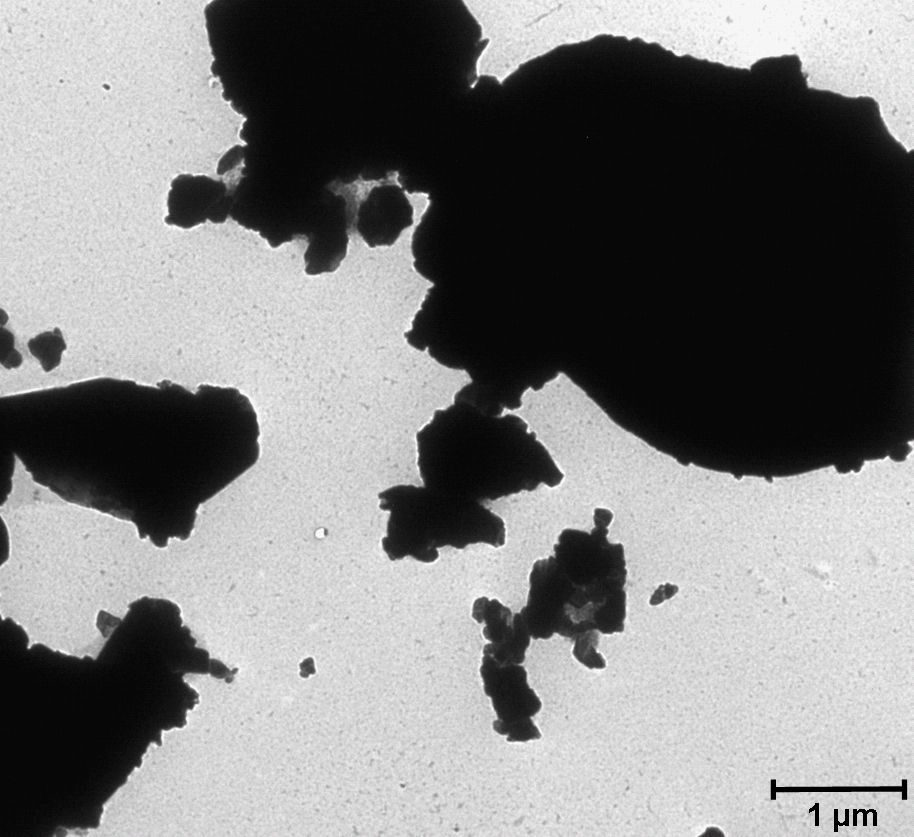


Table S1: Particle Characterization

|  | **Purity [%]** | **Average Size [nm]** | **Size Range**  **[nm]** | **Surface Area [m^2^/g]** | **Crystal Structure** |
| --- | --- | --- | --- | --- | --- |
| TiO_2_ NSP | > 99.0 | 5 | ------ | 210 | anatase |
| TiO_2_ MSP | 99.8 | 1220 | 900-1600 | 4.2 | rutile |
| Polystyrene NSP | 100 | 50 | ------ | 114 | ------ |
| Polystyrene MSP | 100 | 1000 | ------ | 5.7 | ------ |
| Quartz MSP | 97.5 | 2000 | 100-16000 | 6.0 | α-quartz |
